# “Affect of anaerobiosis on the antibiotic susceptibility of *H. influenzae*”

**DOI:** 10.1186/1756-0500-6-241

**Published:** 2013-06-26

**Authors:** Hannah Kendall Smith, Kevin Lee Nelson, Edison S Calaunan, Arnold Lee Smith, Victoria Nguyen

**Affiliations:** 1 Center for Childhood Infections, Seattle Children’ Research Institute, 1900 Ninth Ave Seattle, WA 98101 Seattle, USA; 2 Division of Infectious Disease, Department of Pediatrics, Columbia University, 650 West 168th Street, New York, NY 10032 USA

**Keywords:** *Haemophilus influenzae*, Antibiotic activity, Anaerobiosis

## Abstract

**Background:**

*Haemophilus influenzae* is a human-restricted facultative anaerobe which resides mostly in the oropharynx. The majority of isolates recovered from the throat are unencapsulated commensals (NTHi), but depending on host susceptibility they cause bronchitis, otitis media and on occasion bacteremia and meningitis. Because of the variable oxygen availability in the various niche permitting bacterium replication, the organism must thrive in well oxygenated surfaces, such as pharyngeal epithelium to anoxic environments like the bottom of a Biofilm and in airway mucus. Other reports indicate that *H. influenzae* use aerobic respiration, anaerobic respiration and fermentation to generate ATP. To gain insight in to the activity of several classes of antibiotics against five well-characterized unencapsulated *H. influenzae* in room air, in 5% CO_2 _and under strict anaerobiosis. We also tested for the role of oxidative killing by all cidal antibiotics.

**Results:**

In comparison to room air, testing in 5% CO_2_ had minimal effects on the susceptibility to aminoglycosides, cephalosporins, tetracycline and chloramphenicol*:* the MIC of rifampin and ciprofloxacin increased eight fold with certain strains in 5% CO_2_. All antibiotics, except trimethoprim were cidal under both growth conditions. Aminoglycosides remained bactericidal in a strict anaerobic environment, while a reliable MBC was obtained with trimethoprim only under anaerobic conditions. Kinetic analysis of the cidal action of spectinomycin and tetracycline indicated slower killing anaerobically. An oxidative mechanism for aerobic killing could not be demonstrated.

**Conclusions:**

We conclude that β-lactams, cephalosporins, macrolides, tetracycline’s, aminoglycosides, chloramphenicol, rifampin and ciprofloxacin are bactericidal against five well-characterizes *H. influenzae* in an aerobic and anaerobic environment. The activity of trimethoprim was increased in anaerobic conditions.

## Background

*Haemophilus influenzae* is classified by Bergey’s Manual of Determinative Microbiology (9th Edition, pg 195) as a “nonmotile Gram negative coccobacillus which is a facultative anaerobe having both a respiratory and fermentative type of metabolism” [1]. Multiple studies examing *H. influenzae* gene content or expression under various environmental conditions have suggested that the organism prefers an anaerobic environment, and may use anaerobic respiration and fermentation depending on the local oxygen availability. In well aerated, shaking sBHI broth cultures with *H. influenzae* Rd KW20 incubated at 37°C in room air there is oxygen consumption during logarithmic growth [2]. The partial pressure of oxygen in the media during logarithmic growth averaged 64 Torr during the 120 min of logarithmic growth but varying from 145 to 53 Torr: 64 Torr is approximately an O_2_ concentration of 0.8 mM With the increased consumption of oxygen, there is a reciprocal increase in the pCO_2_; from, < 4 Torr to a peak value of 13.8 Torr at a time when the O_2_ concentration was at its nadir [2]. During the lag and stationary phases the media pO_2_ ranged from 128 to 145 Torr [2]. During overnight incubation of strain Rd KW20 in room air the pH of sBHI decreases from 7.39 to 5.55 indicating fermentation*.* In an aerated and shaken culture, as well as incubation in a BD GasPak, genes of the nitrate operon (*napFDAGHBC)* and the nitrite complex (*nrfABCD)* were detected [3] indicating ongoing anaerobic respiration. As the O_2_ concentration in the media decrease the high affinity *cydAB* is responsible for aerobic respiration. The simultaneous detection of the presence of respiratory and fermentative enzymes in anaerobic and microaerophilic environments indicates that all of the pathways are active in *H. influenzae.*

Others studying *H. influenzae* colonization mechanisms found that strain Eagan and a mutant deficient in MnSOD both grew best at the lowest oxygen supply rate tested, an environment supplying 3 mMol O_2_/liter /hr [4]. Although the MnSOD mutant had decreased colonization capacity of the infant rat nasopharynx, it invasive capacity was unchanged, suggesting a preference for anaerobic or micro aerobic lifestyle after invasion. Studies [5] of the anoxic redox control two-component system, ArcAB indicated that the loss of a functional ArcA response regulator decreased the serum resistance of the type b strain. They found that fumarate reductase was more highly expressed in the *arcA* + parent, while L-Lactate dehydrogenase was derepressed in the *arcA* mutant. Unfortunately the details of the techniques to create an anaerobic environment was filling flasks and sealing them making the environment micro aerobic.

Nontypeable *H. influenzae* form biofilms in the middle ear of children with otitis media [6], a finding that has been reproduced in animal models of otitis media [7]. Bacterial metabolism is not uniform in all portions of the Biofilm with the basilar portion being hypoxic. One explanation for the antibiotic resistance of bacterial biofilms is that the basilar portions are anoxic [8]. Thus understanding the response of *H. influenzae* to antibiotics in an anoxic environment will clarify the role of ambient oxygen concentration on antibiotic activity.

It has been recently proposed that all classes of bactericidal antibiotics kill *E. coli* via an oxidative mechanism, but bacteriostatic antibiotics do not elicit an oxidative respiratory burst [9]. It is not certain how a cidal antibiotic increases aerobic respiration, particularly in a bacterium such as *Haemophilus influenzae* which has uses multiple mechanisms to generate ATP depending on the O_2_ availability. Since all antibiotics which have been tested, except trimethoprim, appear cidal against *H. influenzae* we determined the activity of multiple antibiotics against a well-defined panel of *H. influenzae* in room air, in 5% CO_2_ and in a Coy anaerobic chamber. The antibiotics were chosen to represent different classes and modes of action and were not chosen to inform clinicians. This data should not be used for clinical decisions.

We examined five *H. influenzae* strains whose genome has been sequenced and annotated so that additional insight into antibiotic cidal mechanisms can be explored. We found that all classes of antibiotics were bactericidal and could not detect an oxidative process in bacterial killing.

## Results

Using the aerobic and anaerobic techniques described above we first determined the susceptibility of the reference *H. influenzae* strain for testing antibiotic susceptibility, ATCC 49766 to ampicillin, chloramphenicol and tetracycline. With ampicillin the aerobic MIC/MBC was 0.15/5.0 and 1.5/4.0 μg/ml anaerobically, the aerobic chloramphenicol MIC/MBC was 0.2/ 2.6 and the anaerobic MIC/MBC was 0.4/3.0 μg/ml. With tetracycline the MIC/MBC was 0.25/1.0 μg/ml aerobically and 0.30/2.20 μg/ml anaerobically. These results indicated that anaerobic susceptibility testing with NTHi whose genome sequence is available was feasible. Recognizing that the intrinsic error of MIC testing is 100% [10], we emphasized only those results which were more than double, or 50% of the value obtained in room air.

### Aerobic susceptibility

The aerobic susceptibility of the sequenced isolates is shown in Table 1 and Table 2 while Table 3 and Table 4 depict the susceptibility in 5% CO_2_. The MIC and MBC for the aminoglycosides was slightly increased in the presence of 5% CO_2_ (Table 3 and Table 4). With strain Rd KW20 the erythromycin MBC increased six-fold when tested in the presence of 5% CO2 (compare Table 2, versus Table 4). All tested strains had an increase in the MIC and MBC with ciprofloxacin (Table 2 versus Table 4).

**Table 1:** Aerobic susceptibility in air

**H. influenzae**	**μg/ml**													
	**Amp**		**Tet**		**Spec**		**Tmp**		**Kan**		**Tobr**		**Cam**	
	MIC	MBC	MIC	MBC	MIC	MBC	MIC	MBC	MIC	MBC	MIC	MBC	MIC	MBC
Rd KW20	0.33	0.54	0.29	2.31	22.5	22.5	5.25	225	1.40	1.40	0.70	2.8	0.18	0.26
R2846	0.44	0.50	0.20	2.31	4.22	8.44	1.98	113	0.70	1.40	0.35	1.05	0.26	0.80
R2866	900	>1800	0.29	1.16	16.88	16.88	0.88	113	1.40	1.40	0.70	1.41	0.18	0.70
R3642	675	>1800	0.29	2.31	5.63	14.07	450	1800	1.06	2.11	0.53	0.70	0.14	1.05
R3661	0.33	0.59	0.29	3.72	16.85	16.85	17.58	1800	1.41	2.80	0.70	1.75	0.18	1.05

*produces TEM β-lactamase.

**Table 2:** Aerobic susceptibility in air

**H. influenzae**	**μg/ml**								**ng/ml**			
	**Cfrx**		**Cclr**		**Clar**		**Erm**		**Rif**		**Cip**	
	MIC	MBC	MIC	MBC	MIC	MBC	MIC	MBC	MIC	MBC	MIC	MBC
Rd KW20	0.51	3.00	11.72	5.63	5.63	16.88	0.48	1.93	70.0	70.0	0.71	3.94
R2846	0.68	1.56	11.72	15.94	0.26	1.41	0.26	0.44	26.5	70.0	2.25	4.50
R2866	0.72	1.56	15.94	24.37	2.81	5.63	0.26	0.44	70.0	105.5	1.69	3.10
R3642	1.10	2.81	15.94	30.94	2.81	11.25	0.35	0.97	70.0	105.5	3.38	3.38
R3661	0.85	4.07	12.19	14.06	4.22	22.50	0.53	1.41	35.0	70.0	1.41	4.50

*Amp* ampicillin, *Tet* tetracycline, *Cip* ciprofloxacin, *Clar* clarithromycin, *Cfrx* cefuroxime, *Spec* spectinomycin, *Cclr* Cefaclor, *TMP* trimethoprim, *Rif* rifampin, *Kan* Kanamycin, *Tobr* tobramycin, *Cam* chloramphenicol, *Erm* erythromycin.

**Table 3:** **Aerobic susceptibility in 5% CO**_**2**_

**H. influenzae**	**μg/ml**													
	**Amp**		**Tet**		**Spec**		**Tmp**		**Kan**		**Tobr**		**Cam**	
	MIC	MBC	MIC	MBC	MIC	MBC	MIC	MBC	MIC	MBC	MIC	MBC	MIC	MBC
Rd KW20	1.17	1.41	0.35	5.63	22.5	33.85	9.10	338	4.22	5.63	2.82	4.22	0.27	0.8
R2846	0.44	0.50	0.32	0.94	10.3	18.8	1.10	84.4	1.41	4.22	1.41	2.81	0.27	0.8
R2866	1350	>1800	0.32	2.81	26.3	33.8	0.88	113	1.4	1.4	2.11	2.81	0.27	0.8
R3642	900	>1800	0.25	0.32	5.63	14.1	788	1800	1.41	4.22	1.41	2.11	0.27	1.4
R3661	1.41	2.81	0.29	2.83	18.8	22.5	4.39	1575	2.81	5.63	2.11	4.22	0.35	1.4

**Table 4:** **Aerobic susceptibility in 5% CO**_**2**_

***H. influenzae***	**μg/ml**								**ng/ml**			
	**Cfrx**		**Cclr**		**Clar**		**Erm**		**Rif**		**Cip**	
	MIC	MBC	MIC	MBC	MIC	MBC	MIC	MBC	MIC	MBC	MIC	MBC
Rd KW20	2.11	2.34	15.47	23	18.58	30	3.26	11.69	122.5	140	8.29	10.17
R2846	1.14	4.22	11.7	18.7	0.35	14.10	0.49	0.57	70.0	87.5	8.29	12.04
R2866	1.41	2.81	15.94	24.4	6.57	11.25	3.53	0.57	157	157	16.58	16.58
R3642	1.88	5.63	23.4	30.9	7.5	30.0	0.35	1.41	87.5	105	10.6	16.58
R3661	2.34	5.63	15.9	18.75	11.25	30.0	0.61	1.10	87.5	105	16.58	20.3

*Amp* ampicillin, *Tet* tetracycline, *Cip* ciprofloxacin, *Clar* clarithromycin, *Cfrx* cefuroxime, *Spec* spectinomycin, *Cclr* Cefaclor, *TMP* trimethoprim, *Rif* rifampin, *Kan* Kanamycin, *Tobr* tobramycin, *Cam* chloramphenicol, *Erm* erythromycin.

### Anaerobic susceptibility

Under anaerobic conditions using sBHI with 0.1% ferric nitrate the MBC for tetracycline increased with all strains except with strain R2866, with the values not significantly different with incubation in either environment. Anaerobic incubation had the most dramatic affect on the susceptibility to trimethoprim. In room air or 5% CO_2_ the MBC was difficult to measure accurately: the values reported met the criteria of 99.9% kill, but there was poor reproducibility among the replicates. The MBC value of 1800 μg/ml was the highest trimethoprim concentration tested with the MIC not significantly different except with strain R3932 (anaerobically adapted R3642. Under anaerobic conditions the trimethoprim end-points could easily be identified with all strains except R3392 (anaerobically adapted R3642). In room air the MIC was 450 μg/ml and increased on average one well to 788 μg/ml with the MBC being 1800 μg/ml in room air and 5% CO_2_. Current reviews indicate that bacteria grown in an anaerobic environment are resistant to aminoglycosides as they do not have the required respiration to facilitate drug uptake [11]. However, kanamycin and tobramycin maintained their bactericidal activity under anaerobic conditions, with the MIC and MBC values in the clinical susceptibility range for all strains. The results of the anaerobic susceptibility testing are shown in Table 5 and Table 6.

**Table 5:** Anaerobic susceptibility

**H. influenzae**	**μg/ml**													
	**Amp**		**Tet**		**Spec**		**Tmp**		**Kan**		**Tobr**		**Cam**	
	MIC	MBC	MIC	MBC	MIC	MBC	MIC	MBC	MIC	MBC	MIC	MBC	MIC	MBC
R3929	0.53	4.22	0.18	15.9	25.31	26.72	3.52	42.2	1.06	2.11	1.06	2.81	0.53	2.81
R3931	1.41	4.22	0.35	2.11	18.28	45.0	0.88	5.25	3.17	18.7	4.13	6.56	0.53	2.81
R3930	1575	1800	0.27	1.05	2.11	8.44	0.88	84.4	1.23	2.46	1.23	2.81	0.53	2.81
R3932	1350	1800	0.35	2.81	1.46	2.34	562	675	1.29	1.64	1.28	1.87	0.70	2.81
R3393	1.76	4.22	0.28	8.44	9.92	33.75	3.5	28.10	1.99	3.28	1.64	3.75	0.53	2.81

**Table 6:** Anaerobic susceptibility

**H. influenzae**	**μg/ml**								**ng/ml**			
	**Cfrx**		**Cclr**		**Clar**		**Erm**		**Rif**		**Cip**	
	MIC	MBC	MIC	MBC	MIC	MBC	MIC	MBC	MIC	MBC	MIC	MBC
R3929	0.35	1.78	22.50	45	8.44	45.0	0.35	1.06	33.8	67.5	3.7	5.98
R3931	0.68	1.56	11.25	45	11.25	33.75	0.53	1.41	78.8	123.8	7.0	7.14
R3930	0.48	1.05	16.88	45	2.81	16.88	0.35	0.88	107	315	4.4	7.39
R3932	1.10	19.69	8.44	45	11.25	33.75	0.35	1.41	14.1	28.1	15.5	28.48
R3933	1.23	7.51	16.88	45	11.25	33.75	0.35	1.41	56.3	135	6.70	8.79

*Amp* ampicillin, *Tet* tetracycline, *Cip* ciprofloxacin, *Clar* clarithromycin, *Cfrx* cefuroxime, *Spec* spectinomycin, *Cclr* Cefaclor, *TMP* trimethoprim, *Rif* rifampin, *Kan* Kanamycin, *Tobr* tobramycin, *Cam* chloramphenicol, *Erm* erythromycin.

Strains R2866 (R3930 for the anaerobic adapted derivative) and R3642 (R3931 for the anaerobically adapted derivative) have a TEM-like β-lactamase and are resistant to ampicillin, and as expected they are susceptible to Cefaclor and cefuroxime (Table 6) Of the two cephalosporin’s tested, Cefaclor was the least potent in all of the environments.

### Antibiotic cidal activity

**Figure 1:**
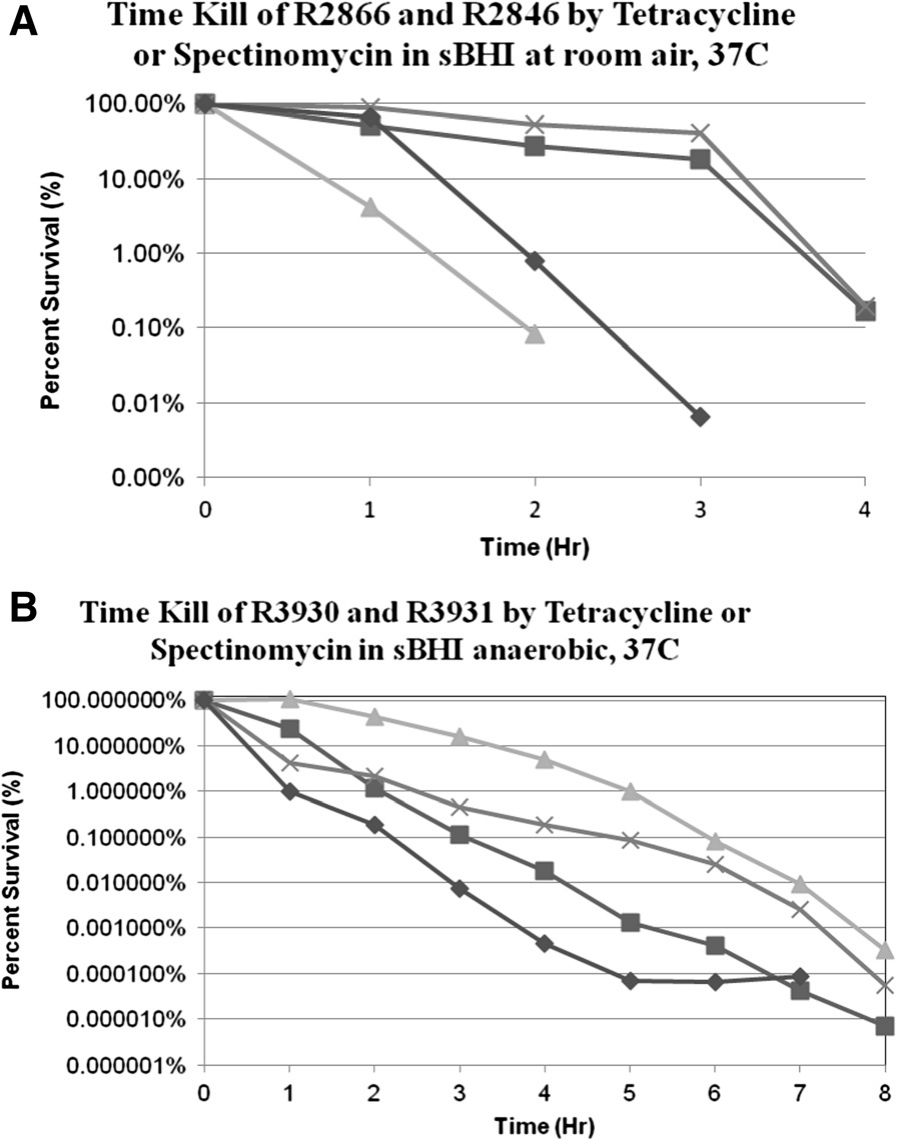
**A. Strain R2866 was incubated in room air with tetracycline at 2 μg/ml (■), or spectinomycin at 20 μg/ml (▲) with the number of CFU determined on aliquots obtained hourly.** Strain R2846 was incubated with 3 μg/ml tetracycline (♦) or spectinomycin at 8 μg/ml (×) with the surviving CFU determined on aliquots obtained hourly. **B**. Strain R2866 was incubated anaerobically with tetracycline at 2 μg/ml (■), or spectinomycin at 20 μg/ml (▲) with the number of CFU determined on aliquots obtained hourly. Strain R2846 was incubated with 3 μg/ml tetracycline (♦) or spectinomycin at 8 μg/ml (×) with the surviving CFU determined on aliquots obtained hourly.

Determining the rate of bacterial killing by antibiotics is a sensitive way of testing antibiotic activity. We chose tetracycline and spectinomycin concentrations which were approximately twice the MBC determined with the micro plate assay. With incubation in room air there was killing of 99.9% of the initial inoculum in four hours with each strain with each antibiotic (Figure 1A). Anaerobic killing by tetracycline was faster with strains R3931 (adapted R2846) and R3930 (adapted R2866) at concentrations which were approximately twice the anaerobic MBC, with the 99.9% kill being achieved at 3 to 4 hours (Figure 1B). With strain R3930 (anaerobic R2866) it took 6 hours before 99.9% of the inoculum was killed by 20 μg/ml spectinomycin (Figure 1B).

### Oxidative cidal mechanism

**Figure 2:**
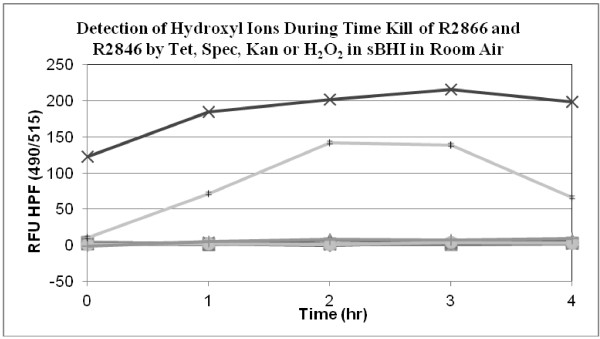
**Hydroxyl ions were detected during an aerobic time-kill assay in room air by measuring the fluorescence at 516**_**nm **_**of. 3′ (p-hydroxyphenyl), fluorescein (HPF, 5 μM)v** Positive controls consisted of 1 mM H_2_O_2_ added to the media with strain R2866 (×) and R2846 (‡). Hydroxyl ions were not detected during incubation of R2866 with 2 μg/ml tetracycline (■) or Kanamycin at 1.5 μg/ml (▲) or with R2846 in media containing tetracycline at 3 μg/ml (•) or Kanamycin at 1.5 μg/ml (▼).

We could not detect hydroxyl ion during the killing of strains R2846 or R2866 by tetracycline, kanamycin or spectinomycin in room air at 37°C with 5 μM 3′(p-hydroxyphenyl) fluorescein (HPF) (Figure 2). As a positive control we conducted a killing curve with 1 mM H_2_O_2_: there was 99.9% killing of both strains within 1 hour of incubation. We also used 3′(p-aminohenyl) fluorescein and could not detect hydroxyl ion during the cidal activity. The killing experiments were repeated with tetracycline, Kanamycin or spectinomycin in the presence of 0.5 mM 2,2′-dipyridyl, an iron chelator which was added simultaneously with antibiotics: Dipyridyl alone had no affect on the rate of loss of viability eliminating a hydroxyl mediated Fenton reaction, a key step in the Collins hypothesis. Additionally we repeated the killing experiments with the same antibiotics in the presence of 150 mM thiourea which quenches hydroxyl ion as it is formed, and found no effect.

### Membrane permeability

**Figure 3:**
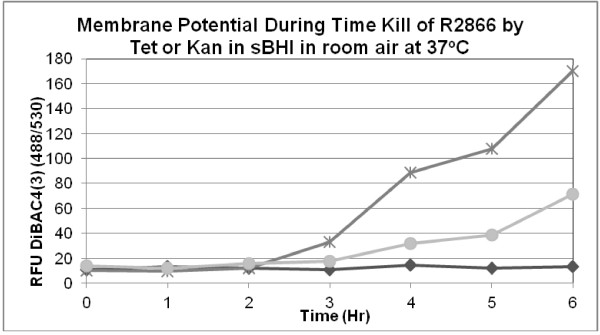
**Strain R2866 was incubated in room air with Kanamycin at 1 μg/ml (×) or tetracycline at 2 μg/ml (♦) in sBHI containing 10 μM bis(1,2-dibutylbarbituric acid) trimethine oxonol [DiBAC**_**4nm**_**(3)] and the fluorescence measured at 530.** Strain R2866 gradually lost membrane permeability during the incubation (●).

The current model for aminoglycosides action is its binding to a site in the ribosome which causes misreading of the mRNA leading to misfolded proteins. These proteins accumulate in the periplasmic space and outer membrane creating channels that permit a larger influx of the aminoglycoside [12]. We found that outer membrane permeability of strain R2866 increased during the cidal action of kanamycin at 1 μg/ml; there was no detectable change in permeability during cell killing with tetracycline at 2 μg/ml (Figure 3). Tetracycline prevents the association of aminoacyl tRNA with the ribosome, which stops protein synthesis [13] and has no immediate effect on the cell membrane. We had previously shown that therapeutic tobramycin concentrations caused mistranslation in aminoglycoside susceptible *H. influenzae*[14].

## Discussion

### Anaerobic growth

In our prior studies on the anaerobic growth of *H. influenzae* strain Rd KW20 we used a disposable GasPak (Becton Dickson) anaerobic chamber after bubbling N_2_ through the media and monitoring anaerobiosis with the GasPak colorimetric indicator. In that study [3] after 24 hours of growth the bacterial density ranged from 1.9 × 10^9^ to 6.5 x 10^9^ CFU/ml in both microaerophilic and “anaerobic” environments. The starting pH of sBHI broth was 7.34 ± 0.12 (n = 9) while after microaerophilic incubation it was 5.97 ± 0.15 (n = 4), after incubation in the anaerobic chamber it was 4.69 ± 0.59 (n = 6).The majority of the proteins detected were present under both growth conditions [3]. There was slightly more acetate, 0.60 mM in the media after anaerobic growth, compared to micro aerobic growth 0.46 mM. This data suggests that *H. influenzae* uses fermentation pathways in both growth environments however the acetate concentration was equivalent with incubation in both environments: 0.46 mM aerobic and 0.60 mM after anaerobic growth [3].

In *H. influenzae* genes, *fnr* and *arcA,* which are transcriptional regulators homologous to *E. coli* genes which modify expression of other genes involved in growth in environments with varying oxygen availability. Deletion of *arcA* increased the expression of enzymes in the respiratory chain and TCA cycle when grown under micro aerobic conditions [15]. In *H. influenzae* having mechanisms which combat oxidative and nitrosative stress are important for its life cycle [16,17]. In addition, *fnr* is required for defense against nitric oxide donors [18]. *H. influenzae* with inactivated *fnr* were unable to metabolize nitrite added to the media indicating that *nrfABCD* was not functional. In a study of the defense of nontypeable *H. influenzae* against reactive nitrogen species Harrington et al. [18] found that placing 7.5 ml of media in a 60 ml DeLong flask resulted in an oxygen saturation (ie dissolved oxygen concentration) of 80%. This corresponds to an O_2_ concentration of 5.7 mg/L or 0.178 mM. This concentration is micro aerobic [19] as the oxygen concentration in well aerated water at 25°C is at 8 mg/L (0.25 mM). However, the micro aerobic condition completely suppressed the expression of a *nrf* fusion [18]. When the Delong flask was filled with 60 ml the oxygen saturation was 38%, 2.6 mg/L or 0.081 mM and the *nrf* fusion was expressed over nine fold greater even though strict anaerobiosis was not achieved.

In silico reviews of *H. influenzae* metabolism concluded that the gene content favored an anaerobic environment [20]. In the study integrating enzyme activity and proteomics, periplasmic nitrate reductase (*napB)* and the cytoplasmic nitrite Reductase (*nrfA)* we found that both enzymes were expressed in anaerobic and micro aerobic growth [2]. However, in the current studies we found that the addition of 10 mM KN0_3_ or 10 mM NaNO_2_ did not increase the growth rate, or the final bacterial density after overnight incubation at 37°C in the anaerobe chamber ( 3–5 x10^9^ CFU/ml) of strains R3930 or R3931.In silico reviews of *H. influenzae* metabolism concluded that the gene content favored an anaerobic environment [20]. In the study integrating enzyme activity and proteomics, periplasmic nitrate reductase (*napB)* and the cytoplasmic nitrite Reductase (*nrfA)* we found that both enzymes were expressed in anaerobic and micro aerobic growth [2]. However, in the current studies we found that the addition of 10 mM KN0_3_ or 10 mM NaNO_2_ did not increase the growth rate, or the final bacterial density after overnight incubation at 37°C in the anaerobe chamber ( 3–5 x10^9^ CFU/ml) of strains R3930 or R3931.

We previously examined the proteome of *H. influenzae* Rd KW20 in late log phase by liquid chromatography coupled with ion trap tandem mass spectrometry [3] and found that the proteome was nearly identical after growth in a Becton Dickson GasPak or in room air. On obtaining these results we serially measured the partial pressure of O_2_ during incubation of 10^3^ CFU/ml in 50 ml of sBHI at 37°C in a 250 ml baffled Erlenmeyer flask agitating the flask at 200 rpm in room air. During the logarithmic growth in sBHI, at an A_650 nm_ between 0.28 and 0.64, the pO_2_ in the media decreased from 125–145 Torr to 53 Torr and remained at 68 Torr during the log phase; but reaching 135 Torr at on entrance to stationary phase, an A_650nm_ of 1.10. Thus the incubation conditions were microaerophilic for the majority of growth during incubation in room air. Additional data measuring enzyme activity in cell lysates as well as organic acids indicated that there was fumarate reductase activity in cells grown in both anaerobic and micro aerobic conditions [2]. Insertional mutagenesis of *frdA* resulted in restricted growth in micro aerobic an anaerobic environment. All of the data indicates that *H. influenzae* Rd KW20 uses classic anaerobic respiration, micro aerobic respiration and fermentative pathways for growth in environments with differing oxygen availability. By using multiple pathways the bacterium can adapt to the different oxygen supply in the human respiratory tract and blood stream. This may represent “hybrid” metabolism in which membrane ArcB located on the membrane mediates changes in gene expression through ArcA when the oxygen supple is too low to penetrate to the cytoplasm and inactivate the direct oxygen sensor, FNR [21].

The marked increase in the potency of trimethoprim under anaerobic conditions (Table 5) suggests that anaerobic growth is more dependent on tetrahydrofolate than aerobic growth. The proteins which were most highly induced in *H. influenzae* strain Rd KW20 with TMP treatment were 5-methylenetetrahydropteroyltrigutamate-homocysteine methyl transferase (*metE*) and serine hydroxymethyl transferase (*glyA)*: both enzymes are involved in the pathway for the synthesis of L-Met, L-Ser, L-Thr and Gly [22].

### Anaerobic killing

One mechanism cited for the decreased activity of antibiotics in an anaerobic environment is the decreased uptake of an aminoglycoside (streptomycin) into the bacterial cell [23].

Testing of a facultative anaerobe such as *E. coli* under strict anaerobic conditions has recently been reported [24,25]. Both of these publications demonstrated that killing by clinically useful concentrations of ampicillin, kanamycin and norfloxacin occurred in Coy chambers. They also found that the hydroxyl ion scavenger, thiourea had no effect on the rate of killing, as we found with *H. influenzae.* Keren et al. also found that testing *E. coli* BW25113 under anaerobic conditions the norfloxacin MICV was 0.25 μg/ml while under aerobic incubation the MIC was 0.125 μg/ml [25].

We found *H. influenzae* strain R3642 (anaerobically adapted R3932) was resistant to ciprofloxacin MIC = 15.5 ng/ml with a MBC of 28.5 ng/ml, whereas aerobically grown strains were susceptible. Others have found that the activity of levofloxacin, a fluoroquinolone with a mechanism of action identical to ciprofloxacin, against *P. aeruginosa* was not affected by anaerobic incubation. We have no explanation for the differing results.

### Hydroxyl ion formation

In contrast to the report of the Collins group which showed that 5 μg/ml of kanamycin killed 99.9% of *E. coli* MG1655 in three hours [9], Keren et al. found that kanamycin at 10 μg/ml killed 90% of *E. coli* BW25113 in the same duration of incubation [25]. We could detect hydroxyl ion during the killing of *H. influenzae* by 1 mM H_2_O_2_(Figure 3), but not during killing by tetracycline and spectinomycin.

These studies do not find evidence for oxidative killing of five well-characterized strains of *H. influenzae* and are in agreement with two recently published papers demonstrating the absence of oxidative killing of *E. coli*[24,25].

## Conclusions

We conclude that β-lactams, cephalosporins, macrolides, tetracycline’s, aminoglycosides, chloramphenicol, rifampin and ciprofloxacin are bactericidal against five well-characterizes *H. influenzae* in an aerobic and anaerobic environment. The activity of trimethoprim was increased in anaerobic conditions.

## Methods

### Antibiotics

Table 7 lists the antibiotics used, their supplier and catalog number. Bacteria were stored at −80°C in sterile skim milk, and were cultured at 37°C in 5% CO_2_ on chocolate agar supplemented with 1% (v/v) IsovitaleX (Becton Dickinson and Co., Franklin Lakes, NJ) or in Difco brain heart infusion broth (Becton Dickinson and Co.) supplemented (sBHI) with hemin HCl (10 μg ml^-1^; Sigma Chemical Co., St. Louis, MO) and β-NAD^+^ (10 μg ml^-1^; Sigma) and with agar (Bacto-Agar, Difco) for solid sBHI plates. To detect hydroxyl ion we used 3′(p-amino phenyl fluorescein) (APF, Invitrogen) at 5 μM, while outer membrane integrity was assessed with bis(1,2-dibutylbarbituric acid) trimethine oxonol [DiBAC_4_(3)] also at 10 μM.

**Table 7:** Antibiotics used

**Antibiotic**	**Catalog number**	**Lot number**	**Vendor**
Ampicillin	BP1760-25	965826	Fisher Scientific
Cefaclor	SI-114 8A	XY5511 AMX	Eli Lilly
Cefuroxime	C4417-1G	110M0794V	Sigma
Chloramphenicol	C-0378	44H0560	Sigma
Ciprofloxacin HCl	86393-320	9867	Miles laboratories
Clarithromycin	C4502	n/a	LKT Laboratories
Erythromycin	329815	3415B88	EMD Millipore
Kanamycin Sulfate	K4000-36	n/a	Sigma
Rifampicin	557303	644293	Calbiochem
Spectinomycin Sulfate	S-9137	81 K12755	Sigma
Tetracycline HCl	T-3383-25	20 K1279	Sigma
Tobramycin	T-1783	124 K0948	Sigma
Trimethoprim	T-7883	27H1015	Sigma

### Bacteria

We first tested the aerobic susceptibility of the ATCC reference strain, *H. influenzae ATCC* 49766 with ampicillin, tetracycline and chloramphenicol with the microtiter plate system to verify the reliability of the technique before examining the test strains. The five test strains were *H. influenzae* Rd KW20 [26]; R2866, a nontypeable strain isolated from the blood of a normal infant with meningitis [27]; R2846 identified as strain 12 on primary isolation from middle ear exudate [28]. Strain R3642 originally identified as 86-028NP was isolated from the nasopharynx of a child with otitis media [29]. R3661 was isolated from middle ear fluid and sequenced by D. Dyer at the University of Oklahoma identified there as 3224A (http://www.microgen.ouhsc.edu). We annotated the R3661 genome with Glimmer v3.02 (http://www.cbcb.umd.edu/software/glimmer/) as we had done with strains R2846 and R2866. Our annotation required that on BLASTn searches homology to other orfs, usually *E. coli* to be 80% or more and the predicted protein mass be at least 75% identical to the homolog. The genomic sequence of these of the five test strains is available at NCBI http://www.ncbi.nlm.nih.gov/genome/genomes/165?subset=.

We chose these five strains as they were sequenced to high coverage, and annotated by hand with reference to biochemical functions when available. With reliable genomic data future experiments concerning the antibiotic activity under anaerobic conditions can be investigated. We did not seek to determine the clinical utility of the antibiotics tested.

All strains were stored at −70°C in skim milk and cultured at 37°C over night in 5% CO_2_ on chocolate agar (aerobic) or sBHI agar (anaerobic) prior to use. Antibiotic susceptibility was determined in BHI broth supplemented with β-NAD^+^ and hemin chloride both at 10 μg/ml (sBHI broth).

### Aerobic susceptibility

We used the technique specified in the CLSI monograph for testing aerobic bacteria (M07-A9) adapted to a microtiter format as previously described [30] with an inoculum of 3 to 5 x 10^5^ CFU/ml: 150 μL of BHI broth supplemented with hemin Cl and β-NAD + (sBHI) was inoculated into each well of a 96 well round bottom Nunc Polystyrene plate (Sigma Cat# P5366). The plate was covered with a lid and incubated at 37°C in room air for 18 to 24 hours. Although the CLSI monograph M07-A8 indicates that testing of *H. influenzae* antibiotic susceptibility be performed in ambient air, it also notes that certain fastidious organisms be tested in 5% CO_2_. We therefore also tested the antibiotic susceptibility in 5% CO_2_.The plates were read in a Spectra Max 190 spectrophotometer with the A_600nm_ of the initial inoculum was 0.089 to 0.124 with overnight growth in antibiotic-free wells giving an average A_600nm_ of 0.313 ± 0.741. Converting the A_600nm_ in the microtiter plate to a 1.0 cm light path an A_600nm_ of 0.313 in the microtiter plate is equal to A_600nm_ of 0.865 in a spectrophotometer with a 1 cm light path or ~3 x10^9^ CFU/ml. The MIC was defined as the antibiotic concentration with an A_600nm _< 0.10, an approximate 90% kill, while subculture of the wells with antibiotic concentrations greater than the MIC permitted calculation of the MBC, a 99.9% kill. Each strain was tested in duplicate and each run was repeated three times, with the mean value of MIC and MBC reported in the tables.

### Anaerobic susceptibility

Because of the variation in the time required for the BBL GasPak system to reach an O_2_ concentration of 0.5% [31] we performed the anaerobic experiments in a Coy anaerobic chamber with an O_2_ concentration of < 0.002 ppm but containing 5% CO_2_. Growth of the five anaerobically adapted *H. influenzae* in sBHI broth containing 0.1% ferric nitrate at 37°C in the Coy chamber increased from a starting A600_nm_ of .0045 ± 0.008 to an average of 0.449 ± 0.060. We adapted CLSI protocol M11-A7 (2007) to the 96 well microtiter plate used for aerobic testing. The test bacteria were passed twice on sBHI agar incubating each subculture overnight at 37°C in the anaerobic chamber. After obtaining good anaerobic growth, the anaerobically adapted strains were renumbered; Rd KW20, R3929; R2866, R3930; R2846,R3931; R3642, R3932 and R3661, R3933 and frozen at −70°C in skim milk. The anaerobically adapted strains were thawed in the Coy chamber, plated on sBHI agar and incubated overnight in the Coy chamber. The bacteria were harvested from these plates and added to sBHI broth which had been stored overnight in the Coy anaerobic chamber at 37°C containing 5% CO_2_. The O_2_ concentration was < 0.002 ppm throughout the incubation. MIC and MBC were determined as described for aerobic incubation. The microtiter plates were removed from the Coy at the end of anaerobic incubation and the A_600nm_read in the spectrophotomer.

### Aerobic killing kinetics

*H. influenzae* strains R2846 and R2866 were grown overnight on sBHI agar incubated at 37°C in 5% CO_2_ and harvested into sBHI broth. The bacterial density was adjusted to 0.2 at 600_nm_, approximately 10^8^ CFU/ml and 1 ml of the bacterial suspension was placed in a 125 ml baffled Erlenmeyer flask containing 10 ml of sBHI; antibiotics were added and incubated at 37°C in room air with shaking at 200 rpm. Aliquots were removed at zero time, 1, 2, 3 and 4 hours of incubation, diluted and plated on sBHI agar and incubated overnight at 37°C in 5% CO_2_ to determine bacterial density. The concentration of tetracycline and spectinomycin selected was approximately twice the MBC value determined in the plate assay. To allow for comparison of the activities we expressed the CFU at each time point as a percentage of the starting inoculum. The strains were tested in duplicate and the assay was repeated twice and the averages plotted.

### Hydroxyl radical measurement

To determine if the aerobic killing of *H. influenzae* by bactericidal antibiotics in room air was via the formation of a hydroxyl radical we measured the oxidation of 5 μM hydroxyphenyl fluorescein (HPF) during the cidal action of tetracycline, kanamycin, spectinomycin and H_2_O_2_ with a Becton Dickinson FACS caliber flow cytometer with excitation at 490_nm_ and emission at 515_nm_. We also repeated the bactericidal assay in the presence of 3′(p-amino phenyl fluorescein) (APF) recording fluorescence as described for HPF. We used both strains at a starting density of 2 – 4 x 10^8^ CFU/ml to obtain a reliable signal and subcultured each strain at 1, 2, 3 and 4 hours of incubation in room air at 37°C in sBHI broth agitating the culture at 200 rpm. To determine if iron was involved in the oxidative bactericidal reaction we repeated the bactericidal assay in the presence of 0.5 mM 2, 2′Dipyrdyl and 150 mM thiourea.

### Outer membrane integrity

To confirm that outer membrane integrity was affected during a kanamycin kill curve in room air we added 10 mM bis(1,2-dibutylbarbituric acid) trimethine oxonol (DiBAC_4_(3))to the cidal assay. DiBAC_4_(3) can enter depolarized cells where it binds to hydrophobic molecules. When cells are lose membrane potential they will continuously take up the dye and increase the cells fluorescence with excitation at 530_nm_ and emission maximum at 560_nm_.

### Anaerobic killing kinetics

Strains R3930 and R3931 were harvested from chocolate agar plates after overnight growth in the Coy chamber at 37°C and 5% CO_2_ with an O_2_ concentration < 0.002 ppm, The bacteria were suspended in sBHI broth containing 0.1% ferric nitrate which had been stored anaerobically for a minimum of two hours and adjusting the density to an A600_nm_ of ~0.2. Tetracycline and spectinomycin were added at approximately twice the anaerobic MBC determined in the micro plate reader and incubated at 37°C in the Coy. The flasks were put in the 37°C incubator that is housed in the Coy chamber with the 125 ml baffled Erlenmeyer flask on a magnetic stirrer. After addition of the antibiotics, producing a final volume of 10 ml, an aliquot was taken and additional aliquots were taken from flasks in the Coy chamber after 1, 2, 3, 4, and 6 hours of incubation, diluted and plated on chocolate agar plates which were incubated in 5% CO_2_ at 37°C to determine bacterial density. We assumed that determining bacterial density under these conditions after bacterial killing occurred accurately indicated bacterial density.

### Availability of supporting data

Raw data on individual strains with specific antibiotics and incubation conditions will be made available on request.

## Abbreviations

sBHI: brain-heart infusion broth supplemented with hemin Cl and β-NAD^+^; MIC: Minimum inhibitory concentration; MBC: Minimum bactericidal concentration; Torr: a unit of pressure equal to 1 mmHg or 0.0193 psi; pO2: the partial pressure of oxygen; pCO2: the partial pressure of carbon dioxide gas; mM: millimolar; NTHi: nontypeable *H. influenzae*; MnSOD: manganese-dependent superoxide dismutase.

## Competing interests

The authors declare that they have no competing interests.

## Authors’ contributions

HKS, EC and VN carried out the experiments. KLN designed the experimental protocols and performed certain experiments. ALS conceived the hypothesis, designed certain experiments and authored the manuscript. All authors read and approved the final manuscript.

## Authors’ information

ALS is professor of Pediatrics and Microbiology at the University of Washington School of Medicine. KLN is a senior technician in ALS laboratory. HKS, VN and EC are all technicians in various laboratories.

## References

[B1] Holt JG Krieg NR Sneath PHA Staley JT Williams ST Williams and Wilkins Bergey's Manual of determinative bacteriology 1994 9 195

[B2] Raghunathan A Price ND Galperin MY Makarova KS Purvine S Picone AF Cherny T Xie T Reilly TJ Munson R Jr In silico metabolic model and protein expression of *Haemophilus influenzae* strain Rd KW20 in rich medium OMICS 2004 8 1 25 41 10.1089/153623104773547471 15107235

[B3] Kolker E Purvine S Galperin MY Stolyar S Goodlett DR Nesvizhskii AI Keller A Xie T Eng JK Yi E Initial proteome analysis of model microorganism *Haemophilus influenzae* strain Rd KW20 J Bacteriol 2003 185 15 4593 4602 10.1128/JB.185.15.4593-4602.2003 12867470 PMC165749

[B4] D'Mello RA Langford PR Kroll JS Role of bacterial Mn-cofactored superoxide dismutase in oxidative stress responses, nasopharyngeal colonization, and sustained bacteremia caused by *Haemophilus influenzae* type b Infect Immun 1997 65 7 2700 2706 9199439 10.1128/iai.65.7.2700-2706.1997 PMC175381

[B5] De Souza-Hart JA Blackstock W Di Modugno V Holland IB Kok M Two-component systems in *Haemophilus influenzae*: a regulatory role for ArcA in serum resistance Infect Immun 2003 71 1 163 172 10.1128/IAI.71.1.163-172.2003 12496162 PMC143216

[B6] Hall-Stoodley L Hu FZ Gieseke A Nistico L Nguyen D Hayes J Forbes M Greenberg DP Dice B Burrows A Direct detection of bacterial biofilms on the middle-ear mucosa of children with chronic otitis media JAMA 2006 296 2 202 211 10.1001/jama.296.2.202 16835426 PMC1885379

[B7] Swords WE Moore ML Godzicki L Bukofzer G Mitten MJ VonCannon J Sialylation of lipooligosaccharides promotes biofilm formation by nontypeable *Haemophilus influenzae* Infect Immun 2004 72 1 106 113 10.1128/IAI.72.1.106-113.2004 14688087 PMC343998

[B8] Hall-Stoodley L Stoodley P Evolving concepts in biofilm infections Cell Microbiol 2009 11 7 1034 1043 10.1111/j.1462-5822.2009.01323.x 19374653

[B9] Kohanski MA Dwyer DJ Hayete B Lawrence CA Collins JJ A common mechanism of cellular death induced by bactericidal antibiotics Cell 2007 130 5 797 810 10.1016/j.cell.2007.06.049 17803904

[B10] Andrews JM Determination of minimum inhibitory concentrations J Antimicrob Chemother 2001 48 Suppl 1 5 16 10.1093/jac/48.suppl_1.5 11420333

[B11] Kohanski MA Dwyer DJ Collins JJ How antibiotics kill bacteria: from targets to networks Nat Rev Microbiol 2010 8 6 423 435 10.1038/nrmicro2333 20440275 PMC2896384

[B12] Nichols WW Young SN Respiration-dependent uptake of dihydrostreptomycin by *Escherichia coli.* Its irreversible nature and lack of evidence for a uniport process Biochem J 1985 228 2 505 512 2409962 10.1042/bj2280505 PMC1145009

[B13] Schnappinger D Hillen W Tetracycline’s: antibiotic action, uptake, and resistance mechanisms Arch Microbiol 1996 165 6 359 369 10.1007/s002030050339 8661929

[B14] Levy J Burns JL Mendelman PM Wong K Mack K Smith AL Effect of tobramycin on protein synthesis in 2-deoxystreptamine aminoglycoside-resistant clinical isolates of *Haemophilus influenzae* AntimicrobAgents Chemother 1986 29 3 474 481 10.1128/AAC.29.3.474 PMC180417 3487286

[B15] Wong SM Alugupalli KR Ram S Akerley BJ The ArcA regulon and oxidative stress resistance in *Haemophilus influenzae* Mol Microbiol 2007 64 5 1375 1390 10.1111/j.1365-2958.2007.05747.x 17542927 PMC1974803

[B16] Wong SaA BJ Kidd S Coordinated regulation of stress and virulence in stages of *Haemophilus* pathogenesis Stress response in pathogenic bacteria 2011 33 47 chapter 2

[B17] Harrison A Bakaletz LO Munson RS Jr *Haemophilus influenzae* and oxidative stress Front Cell Infect Microbiol 2012 2 40 22919631 10.3389/fcimb.2012.00040 PMC3417577

[B18] Harrington JC Wong SM Rosadini CV Garifulin O Boyartchuk V Akerley BJ Resistance of *Haemophilus influenzae* to reactive nitrogen donors and gamma interferon-stimulated macrophages requires the formate-dependent nitrite Reductase regulator-activated ytfE gene Infect Immun 2009 77 5 1945 1958 10.1128/IAI.01365-08 19289513 PMC2681730

[B19] Sabra W Kim EJ Zeng AP Physiological responses of *Pseudomonas aeruginosa* PAO1 to oxidative stress in controlled micro aerobic and aerobic cultures Microbiology 2002 148 Pt 10 3195 3202 12368453 10.1099/00221287-148-10-3195

[B20] Tatusov RL Mushegian AR Bork P Brown NP Hayes WS Borodovsky M Rudd KE Koonin EV Metabolism and evolution of *Haemophilus influenzae* deduced from a whole- genome comparison with Escherichia coli CurrBiol 1996 6 3 279 291 10.1016/s0960-9822(02)00478-5 8805245

[B21] Rolfe MD Ocone A Stapleton MR Hall S Trotter EW Poole RK Sanguinetti G Green J Systems analysis of transcription factor activities in environments with stable and dynamic oxygen concentrations Open Biol 2012 2 7 120091 10.1098/rsob.120091 22870390 PMC3411108

[B22] Evers S Di Padova K Meyer M Fountoulakis M Keck W Gray CP Strategies towards a better understanding of antibiotic action: folate pathway inhibition in *Haemophilus influenzae* as an example Electrophoresis 1998 19 11 1980 1988 10.1002/elps.1150191116 9740058

[B23] Schlessinger D Failure of aminoglycoside antibiotics to kill anaerobic, low-pH, and resistant cultures Clin Microbiol Rev 1988 1 1 54 59 3060245 10.1128/cmr.1.1.54 PMC358029

[B24] Liu Y Imlay JA Cell death from antibiotics without the involvement of reactive oxygen species Science 2013 339 6124 1210 1213 10.1126/science.1232751 23471409 PMC3731989

[B25] Keren I Wu Y Inocencio J Mulcahy LR Lewis K Killing by bactericidal antibiotics does not depend on reactive oxygen species Science 2013 339 6124 1213 1216 10.1126/science.1232688 23471410

[B26] Fleischmann RD Adams MD White O Clayton RA Kirkness EF Kerlavage AR Bult CJ Tomb JF Dougherty BA Merrick JM Whole-genome random sequencing and assembly of *Haemophilus influenzae* Rd Science 1995 269 5223 496 512 10.1126/science.7542800 7542800

[B27] Nizet V Colina KF Almquist JR Rubens CE Smith AL A virulent nonencapsulated Haemophilus influenzae J Infect Dis 1996 173 1 180 186 10.1093/infdis/173.1.180 8537657

[B28] Barenkamp SJ Leininger E Cloning, expression, and DNA sequence analysis of genes encoding nontypeable *Haemophilus influenzae* high-molecular-weight surface-exposed proteins related to filamentous hemagglutinin of Bordetella pertussis Infect Immun 1992 60 4 1302 1313 1548058 10.1128/iai.60.4.1302-1313.1992 PMC256997

[B29] Harrison A Dyer DW Gillaspy A Ray WC Mungur R Carson MB Zhong H Gipson J Gipson M Johnson LS Genomic sequence of an otitis media isolate of nontypeable *Haemophilus influenzae* : comparative study with *H. influenzae* serotype d, strain KW20 J Bacteriol 2005 187 13 4627 4636 10.1128/JB.187.13.4627-4636.2005 15968074 PMC1151754

[B30] Doern GV In vitro susceptibility testing of *Haemophilus influenzae*: review of new National Committee for Clinical Laboratory Standards recommendations J Clin Microbiol 1992 30 12 3035 3038 1452681 10.1128/jcm.30.12.3035-3038.1992 PMC270584

[B31] Imhof A Heinzer I Continuous monitoring of oxygen concentrations in several systems for cultivation of anaerobic bacteria J Clin Microbiol 1996 34 7 1646 1648 8784562 10.1128/jcm.34.7.1646-1648.1996 PMC229087

